# SIV infection aggravates malaria in a Chinese rhesus monkey coinfection model

**DOI:** 10.1186/s12879-019-4465-6

**Published:** 2019-11-12

**Authors:** Guangjie Liu, Youjia Li, Li Qin, Yongxiang Yan, Yijian Ye, Yue Chen, Cuizhu Huang, Siting Zhao, Yongchao Yao, Zhong Su, Xiaoping Chen

**Affiliations:** 1 Laboratory of Pathogen Biology, State Key Laboratory of Respiratory Diseases, Center of Infection and Immunity, Guangzhou Institutes of Biomedicine and Health, Chinese Academy of Sciences, 190 Kaiyuan Avenue, Guangzhou Science Park, Guangzhou, 510530 China; 2 Laboratory of Immunobiology, State Key Laboratory of Respiratory Disease, Center of Infection and Immunity, Guangzhou Institutes of Biomedicine and Health, Chinese Academy of Sciences, Guangzhou, 510530 China; 3 Graduate School, University of Chinese Academy of Sciences, Chinese Academy of Sciences, Beijing, China; 4 Shenzhen Institute of Geriatrics, Shenzhen Second People’s Hospital, The First Affiliated Hospital of Shenzhen University, Shenzhen, China; 5 The First Affiliated Hospital, Sun Yat-sen University, Guangzhou, China

**Keywords:** *Plasmodium cynomolgi*, Simian immunodeficiency virus (SIV), Coinfection, Rhesus macaques, Antibody, Cellular immune response, Neopterin, Immune activation, Turnover

## Abstract

**Background:**

The co-occurrence of human immunodeficiency virus (HIV) infection and malaria in humans in endemic areas raises the question of whether one of these infections affects the course of the other. Although epidemiological studies have shown the impact of HIV infection on malaria, the mechanism(s) are not yet fully understood. Using a Chinese rhesus macaque coinfection model with simian immunodeficiency virus (SIV) and *Plasmodium cynomolgi* (Pc) malaria, we investigated the effect of concurrent SIV infection on the course of malaria and the underlying immunological mechanism(s).

**Methods:**

We randomly assigned ten Chinese rhesus monkeys to two groups based on body weight and age. The SIV-Pc coinfection animals (S + P group) were infected intravenously with SIVmac251 eight weeks prior to malaria infection, and the control animals (P group) were infected intravenously with only Pc-infected red blood cells. After malaria was cured with chloroquine phosphate, we also initiated a secondary malaria infection that lasted 4 weeks.

We monitored body weight, body temperature and parasitemia, measured SIV viral loads, hemoglobin and neopterin, and tracked the CD4^+^, CD8^+^, and CD4^+^ memory subpopulations, Ki67 and apoptosis by flow cytometry. Then, we compared these parameters between the two groups.

**Results:**

The animals infected with SIV prior to Pc infection exhibited more severe malaria symptoms characterized by longer episodes, higher parasitemia, more severe anemia, greater body weight loss and higher body temperature than the animals infected with Pc alone. Concurrent SIV infection also impaired immune protection against the secondary Pc challenge infection. The coinfected animals showed a reduced B cell response to Pc malaria and produced lower levels of Pc-specific antibodies. In addition, compared to the animals subjected to Pc infection alone, the animals coinfected with SIV and Pc had suppressed total CD4^+^ T cells, CD4^+^CD28^high^CD95^high^ central memory T cells, and CD4^+^CD28^low^CD95^−^ naïve T cells, which may result from the imbalanced immune activation and faster CD4^+^ T cell turnover in coinfected animals.

**Conclusions:**

SIV infection aggravates malaria physiologically and immunologically in Chinese rhesus monkeys. This nonhuman primate SIV and Pc malaria coinfection model might be a useful tool for investigating human HIV and malaria coinfection and developing effective therapeutics.

## Background

Malaria is a life-threatening infectious disease that is prevalent in sub-Saharan Africa and caused by *Plasmodium* parasites. The most recent statistics show that there were 216 million malaria cases and 0.5 million recorded malaria deaths worldwide in 2016, approximately 90% of which occurred in Africa [1]. Human immunodeficiency virus (HIV) targets and impairs immune defenses against infections and some type of cancers, leading to acquired immunodeficiency syndrome (AIDS). WHO data indicate that approximately 70% of the 36.7 million HIV patients worldwide live in Africa [2]. Given that the endemic regions of *Plasmodium* and HIV infection overlap extensively and that many people are infected, there is an increased risk of coinfection with these two pathogens.

Because these two infections have a profound impact on human health, many studies have been conducted to investigate the potential interactions between them. Although some early studies failed to observe any direct associations between HIV and malaria [3, 4], several recent studies have shown that concomitant HIV infection worsens malaria. The incidence of malaria infection is increased in HIV-endemic regions and in pregnant women [5, 6], and more severe clinical symptoms of malaria occur in HIV^+^ adults with partial immunity to the parasites [7, 8]. Limited clinical and experimental studies showed that impaired immune activation, reduced anti-malaria antibody production and subsequent immunosuppression were associated with an increased frequency of clinical malaria and parasitemia in HIV-infected individuals [9–14]. However, the immunological interaction between these two important infectious diseases is not fully understood.

Previous studies on the interaction between HIV infection and malaria in humans showed disparate results and often reached inconsistent conclusions, probably due to the confounding factors involved in human studies. Nonhuman primates have been used as a suitable model for the study of human malaria HIV infection. Rhesus macaques can be infected by several *Plasmodium* species and by simian immunodeficiency virus (SIV) [15, 16]. *Plasmodium cynomolgi* (Pc) infection in monkeys is an established model for the study of *Plasmodium vivax* infections in humans [17, 18]. SIV infection in Chinese rhesus macaques shows many features that are similar to those of HIV infection in humans [19]. We established a Chinese rhesus macaque coinfection model to study how Pc malaria modulates the course of SIV infection under controlled experimental conditions [20]. Using a similar coinfection model, we investigated the modulatory effects of SIV infection on the course of Pc malaria. We observed that pre-existing SIV infection profoundly modulates the course and severity of primary and secondary blood-stage Pc malaria. In addition, SIV infection alters the humoral immune response, the homeostasis of CD4^+^ T cells and immune activation during the acute phase of Pc malaria, which may lead to impaired immunity and exacerbated malaria.

## Methods

### Animals

Adult Chinese rhesus macaques (*Macaca mulatta*) used in this study, 5–6 years of age, were purchased from a commercial breeding farm (Jiufo Monkey Farm) in Guangzhou, China. All animals were confirmed to be free of *Plasmodium*, B virus, D-type simian retrovirus, simian T-lymphotropic virus type 1 and SIV. The animals were housed at the Non-Human Primate Animal Center of the Guangzhou Institutes of Biomedicine and Health (GIBH). The animal experiments were designed and performed in accordance with the NIH Guide for the Care and Use of Laboratory Animals, and the protocols were approved by the GIBH Institutional Animal Care and Use Committee. Each animal was housed in a separate cage, received standard primate feed and fresh fruit or eggs daily, and had free access to water. The animals were monitored routinely for body temperature, body weight, physical fitness and blood biochemistry. After this experiment, the macaques were included into another coinfection study. They were euthanized when they presented with advanced stages of AIDS; criteria for euthanasia included 15% weight loss in 2 weeks, unresponsive opportunistic infection, persistent anorexia, intractable diarrhea, progressive neurologic signs or other serious illness. Macaques were euthanized via infusion of sodium pentobarbital after anesthesia with ketamine HCl by a veterinarian.

### Pc malaria and SIV infection procedures

**Fig. 1:**
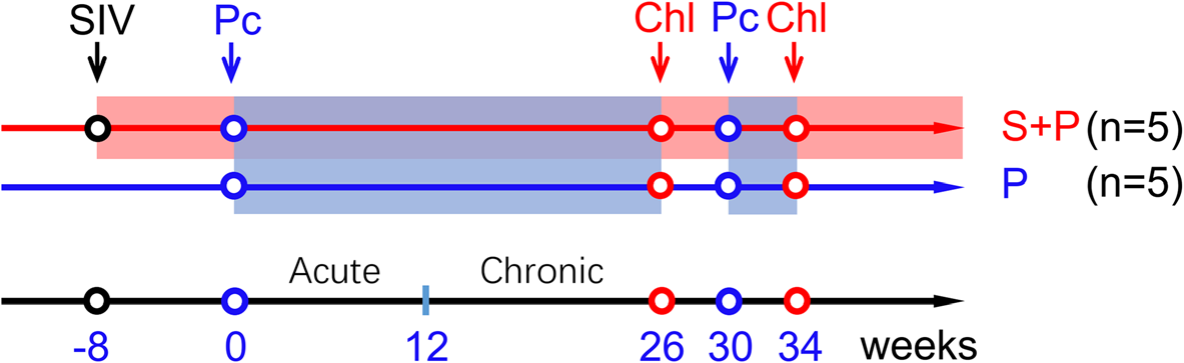
Experimental design for SIV and blood-stage *Plasmodium cynomolgi* (Pc) malaria coinfection in Chinese rhesus monkeys. At week − 8, five monkeys were infected with SIVmac251. At week 0, the SIV-infected monkeys and a group of naïve monkeys (*n* = 5) were inoculated with blood-stage Pc parasites. Primary malaria infection was terminated by a three-day chloroquine phosphate treatment (Chl) at week 26. Four weeks later, all animals were reinoculated with Pc. All monkeys were treated with chloroquine phosphate for 3 days at week 34

Here, we established a coinfection model of SIV and blood-stage Pc malaria parasites in Chinese rhesus monkeys. Ten monkeys were randomly assigned to two groups based on body weight and age, but sex was not considered. The experimental design is shown in Fig. 1. The SIV-Pc coinfection animals (S + P group, *n* = 5; four males and one female) were infected intravenously (i.v.) with 300 TCID_50_ of SIVmac251 eight weeks prior to malaria infection, and the control animals (P group, n = 5; one male and four females) were infected i.v. with only 1 × 10^7^ Pc-infected red blood cells (iRBCs) from a donor rhesus macaque that was infected with blood-stage parasites that were reconstituted from ring-stage cryopreserved stocks of the Pc B strain. The infected animals in the S + P and P groups were treated orally with chloroquine phosphate for 3 days (total dose = 67 mg/kg) at week 26 post-Pc infection to cure malaria. All animals in both groups were reinfected with Pc at week 30 to initiate a secondary malaria infection, and the malaria was cured 4 weeks later, as described above. When the parasitemia reached 15–20% (iRBCs/total RBCs), the animals were administered 10 mg of artesunate (Guilin Pharmaceutical Co. Ltd., Guilin, Guangxi, China) i.v. once to temporarily control Pc replication and prevent death.

Parasitemia was monitored daily by preparation and microscopic examination of blood films stained with Giemsa stain (Sigma-Aldrich, St. Louis, Missouri, USA). Body temperature was measured in the inner ear twice daily with a medical digital ear thermometer.

### Blood collection and preparation

Blood for parasitemia was obtained by pricking a finger or earlobe. Both thick and thin blood films were prepared [21]. Venous blood was incubated at room temperature for 2 h to allow clotting, and serum was carefully aspirated. K2-EDTA anticoagulated venous blood was centrifuged at 1000×g, and the supernatant (plasma) was collected for SIV RNA isolation. K2-EDTA anticoagulated venous whole blood was used for flow cytometry.

### Viral detection

Plasma viral RNA levels were determined using a SYBR Green-based real-time PCR protocol, as previously described [22]. The viral RNA was purified with a QIAamp Viral RNA Mini Kit (Qiagen, Valencia, CA, USA), and real-time PCR was carried out with a one-step QuantiTect SYBR Green RT-PCR Kit (Qiagen, Valencia, CA, USA) on CFX96 Real-Time PCR Detection System (Bio-Rad, Hercules, California, USA).

### Flow cytometry

B cells and T cell subpopulations in peripheral blood were analyzed by flow cytometry. The following fluorescence-conjugated antibodies were used: anti-CD3-PerCP, anti-CD4-FITC, anti-CD4-PE-Cy7, anti-CD4-PerCP (L200), anti-CD8-APC-Cy7 (RPA-T8), anti-CD45-PE (DO58–1283), anti-CD28-FITC (CD28.2), anti-CD95-APC, anti-CD95-PE Cy5 (DX2) and anti-Ki-67-PE (BD Pharmingen, San Diego, CA, USA), anti-CD8-APC and anti-CD8-PE (B9.1) (Immunotech SAS, Marseille Cedex, France). Cell surface marker staining was performed as follows: 50 μl of EDTA-treated whole blood was stained with antibodies at room temperature (RT) for 20 min in the dark, and red blood cells were then lysed in lysis buffer (BD Pharmingen, San Diego, CA, USA) at RT for 10 min, followed by a wash with PBS containing 2% FBS. The number of lymphocytes in peripheral blood samples was determined using the BD TruCOUNT Kit (BD Biosciences, San Jose, CA, USA) according to the manufacturer’s instructions. For intracellular Ki-67 staining, the cells were fixed and permeabilized using the BD Fixation/Permeabilization Solution Kit according to the manufacturer’s instructions, with surface marker staining as described above. Apoptosis and cell death were detected with a PE annexin V apoptosis detection kit (BD Pharmingen, San Diego, CA, USA) according to the manufacturer’s instructions. The stained cells in all assays were stored in the dark at 4 °C and immediately analyzed by flow cytometry on either a FACSCalibur or FACSAria (BD Biosciences, San Jose, CA, USA) system, and the measurements of all samples were completed within 4 h after the staining. The data were analyzed using FlowJo software (Tree Star. Inc., USA).

### ELISA

#### Pc antibodies

Serum levels of Pc-specific antibodies were determined by ELISA. ELISA plates (Nunc A/S, Denmark) were coated with soluble Pc antigens prepared as described previously [23] at a concentration of 6 μg/ml in PBS overnight at 4 °C. The plates were blocked with 1% bovine serum albumin in PBS for 1 h. Individual serum samples were subjected to twofold serial dilution, and 50 μl of each dilution was added to the plate and incubated for 2 h at RT. After extensive washing, a horseradish peroxidase-conjugated goat anti-rhesus IgG (H + L) antibody (SouthernBiotech, Birmingham, AL, USA) was added and incubated at RT for 2 h. Reactivity was visualized with the 3′,3′,5′,5′-tetramethylbenzidine substrate and stopped with 100 μl of a 1 M H_2_SO_4_ solution. OD values were read at 450 nm microplate reader (Bio-TEK, Winooski, Vermont, USA).

#### Neopterin concentration

We determined the neopterin concentration in plasma collected 0, 1, 2 and 5 weeks after Pc inoculation with an ELISA kit (Alpha Diagnostic, San Antonio, Texas, USA) according to the instructions provided in the manual.

### Statistical analysis

GraphPad Prism (Version 6) was used for statistical analysis. Intergroup comparisons were performed using an unpaired t test. Pearson’s correlation analysis was performed to analyze the data for viral load, antibody levels and neopterin levels. Analysis of body weight changes before and after Pc inoculation was performed using multiple comparisons with the Sidak correction after repeated measures two-way ANOVA, and the adjusted *P* value was reported. Data are reported as the mean ± SD. *P* values less than 0.05 were considered statistically significant.

## Results

### Modulation of malaria by concurrent SIV infection

**Fig. 2:**
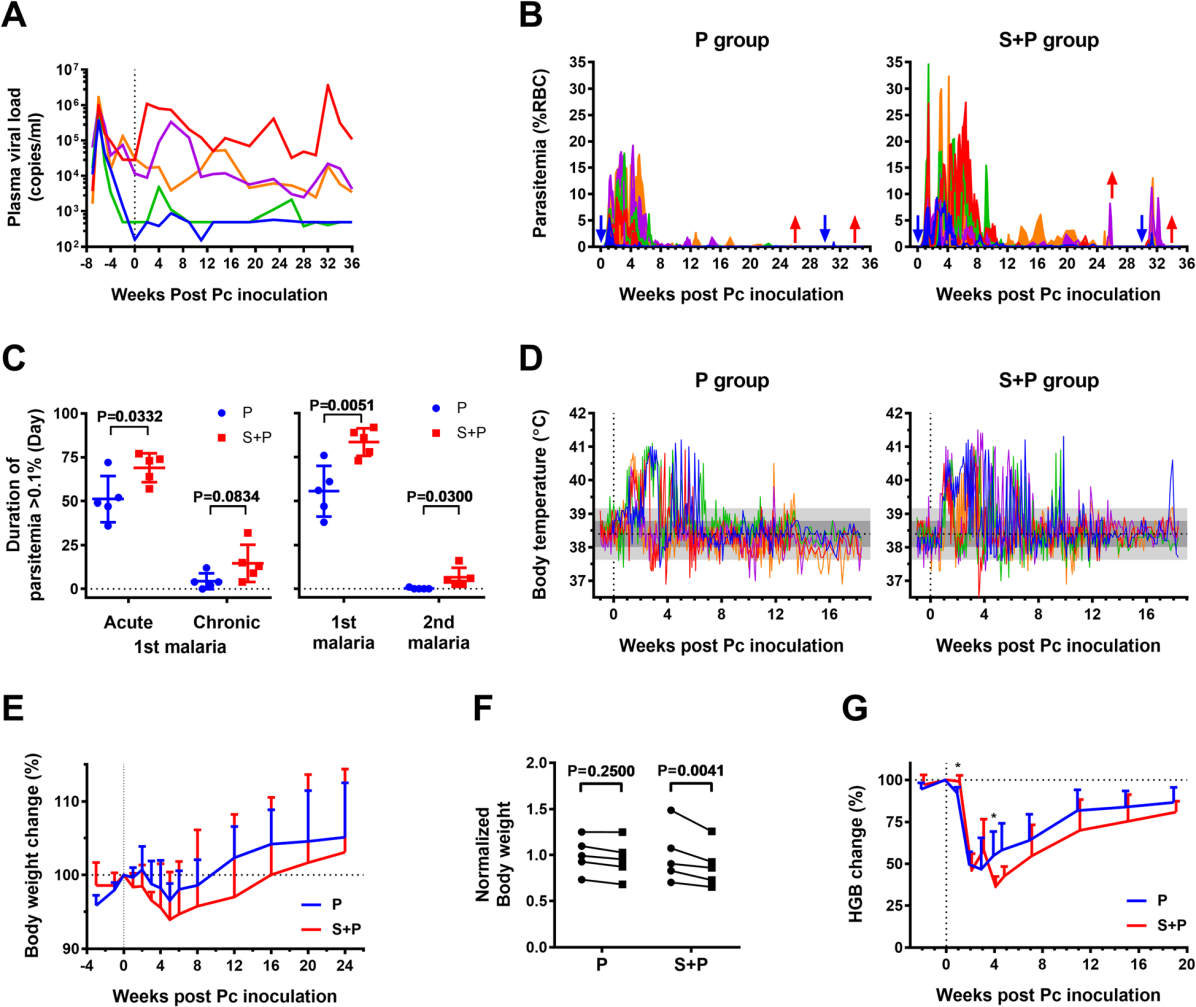
The course and malaria-associated symptoms of Pc-infected and S + P coinfected monkeys. **a** Plasma viral loads of individual SIV-infected monkeys measured by real-time PCR. **b** Parasitemia levels in monkeys singly infected with Pc parasites (P group) or coinfected with SIV and Pc (S + P group). Parasitemia was measured daily by blood smears. Pc inoculation and chloroquine treatment are indicated by blue and red arrows, respectively. **c** Time period when patent parasitemia (> 0.1% RBCs) was detected during the acute (week 0–12) and chronic (week 12–26) stages of primary malaria (left) and following primary and secondary malaria (right). **d** Body temperature changes in the P and S + P groups. Body temperature was measured daily, and data are presented for individual animals. The mean body temperature (38.4 °C) of all animals measured from day − 7 to 0 of Pc infection is shown by a horizontal dotted line. One SD (0.3862 °C) and 2 SDs of the mean body temperature are indicated by the dark gray and light gray zones, respectively. A body temperature higher than 2 SDs of the mean was considered a fever. **e** Body weight changes in the two groups of animals relative to the day of Pc inoculation. **f** The greatest body weight changes in individual animals after Pc inoculation. The values are the ratios of the individual weight to the mean weight of the group before inoculation. The adjusted *P* value was calculated by multiple comparison tests with the Sidak correction, which was performed after repeated measures two-way ANOVA (interaction: *P* = 0.0824, SIV infection: *P* = 0.8164, Pc: *P* = 0.0025). **g** Changes in hemoglobin levels in animals in the P group and S + P group during the first 20 weeks of primary Pc infection. Data presented in C, E, F, and G are the mean ± SD. Unpaired t tests were used, and statistically significant differences are indicated with *(*P* < 0.05) or **(*P* < 0.01)

Five monkeys were successfully infected with SIVmac251. The SIV viral load in plasma was detected 1 week after infection and reached peak levels at 2 weeks after infection (Fig. 2a). Eight weeks after SIV infection, animals in both the S + P and P groups were subjected to Pc infection when the viral loads in the animals in the S + P group decreased to a stable level. All animals in the two groups showed parasitemia that reached peak levels 9–11 days postinoculation. However, the parasitemia of the animals in the S + P group rose more rapidly than the animals in the P group, and three monkeys in the S + P group presented extremely high peaks of parasitemia (C2, 27.2%; C3, 34.6%; and C6, 16.0%). These animals were treated with artesunate on day 11 of Pc inoculation. Four monkeys in the S + P group received 1–3 artesunate treatment(s) 2–7 weeks postinoculation, while only three monkeys in the P group received one treatment 3–4 weeks postinoculation to control Pc parasite replication (Additional file 1: Figure S1). In general, the animals in the S + P group developed higher peak levels of parasitemia (9.3–34.6%) than the animals in the P group (5.3–19.2%) during the acute stage (0–12 weeks) of primary Pc infection (Fig. 2b, Additional file 1: Figure S1). The S + P group animals maintained high levels of parasitemia for a longer duration than the P group animals (Fig. 2b, c). The animals in the S + P group showed higher levels of fever during the course of Pc infection than the animals in the P group (Fig. 2d, Additional file 2: Figure S2, Additional file 3: Figure S3A). The number of days with fever was greater in the S + P group than in the P group, but the difference was not statistically significant (Additional file 3: Figure S3B). The animals in the S + P group showed greater body weight loss following Pc infection than the animals in the P group (Fig. 2e, f, Additional file 3: Figure S3C). The losses in body weight after malaria differed significantly between the two groups. All animals developed severe anemia manifested by a decrease in hemoglobin during the acute and chronic stages of Pc infection, and the difference in hemoglobin levels between the two groups was not statistically significant (Fig. 2g).

All animals were treated with chloroquine at week 26 of Pc infection to terminate malaria and were reinfected with Pc parasites 4 weeks later (Fig. 1). The S + P group animals developed malaria episodes, but all the animals in the P group controlled the parasites to a very low level (parasites could be observed only in thick smear slides) (Fig. 2b, c, Additional file 1: Figure S1).

### Alteration of the humoral immune response to malaria

**Fig. 3:**
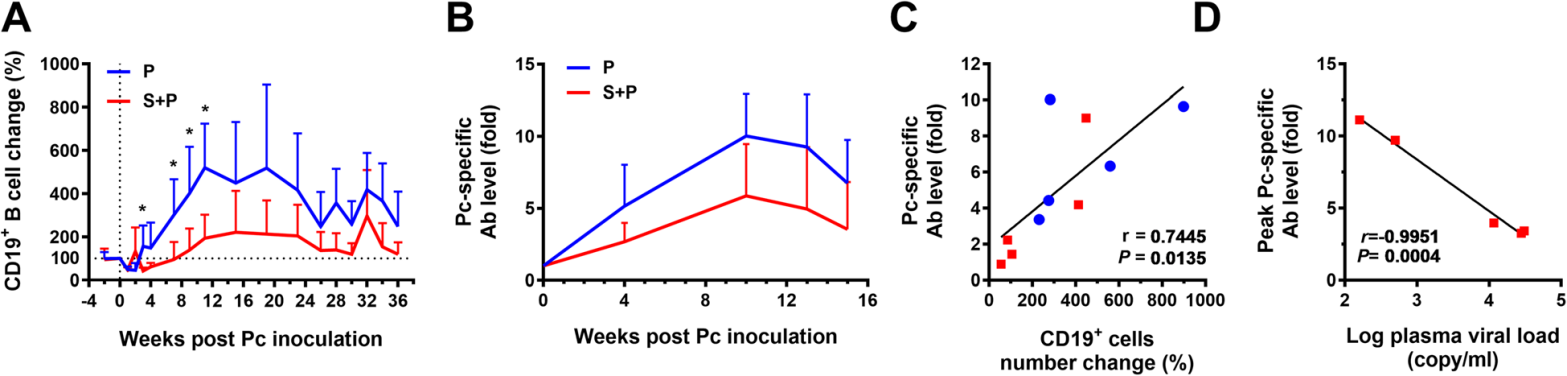
The humoral response to Pc parasites. **a** CD19^+^ B cell numbers in the peripheral blood during the course of Pc malaria. Data are presented as the percent change relative to the values at day 0 of Pc inoculation. **b** Serum levels of Pc-specific antibodies. Data are expressed as the fold increase relative to the level on the day of Pc inoculation. An unpaired t test was performed to analyze the statistical significance of the data presented in A and B, and significant differences are indicated with * (P < 0.05). **c** Correlation between the CD19^+^ B cell count and Pc-specific antibody levels at week 15 of Pc infection. Red square: S + P group, blue circle: P group. **d** Correlation between the logarithm of the viral load on day 0 and the highest Pc antibody levels in five S + P group animals. Pearson r values and *P* values are shown

To determine whether pre-existing SIV infection affects the humoral immune response to Pc malaria, we analyzed the CD19^+^ B cell response and antibody production following Pc infection. The number of B cells in the peripheral blood decreased (day 0 level = 100%) transiently in the first week of Pc infection in the P group and then increased by fivefold until 11 weeks. The S + P group animals showed a delayed and reduced increase in B cells. The B cell number in the S + P group was significantly lower than that in the P group from weeks 3 to 11 after primary Pc inoculation (Fig. 3a). The number of B cells in two groups increased at week 32 (2 weeks after the second Pc infection) and decreased in the following 4 weeks. During the whole course, the B cell number in the P group was higher than the S + P group. Lower levels of Pc-specific antibodies were detected by ELISA in the serum of S + P coinfected monkeys than in the serum of the P group animals (Fig. 3b). The Pc-specific antibody levels were positively correlated with the number of B cells at week 15 of Pc infection (Fig. 3c). We also found that the peak levels of Pc-specific antibodies in the five S + P group animals at weeks 10–13 of Pc infection were negatively correlated with their SIV viral loads at the time of Pc inoculation (day 0) (r = − 0.9951, *P* = 0.0004) (Fig. 3d).

### Responses of the T cell subset

**Fig. 4:**
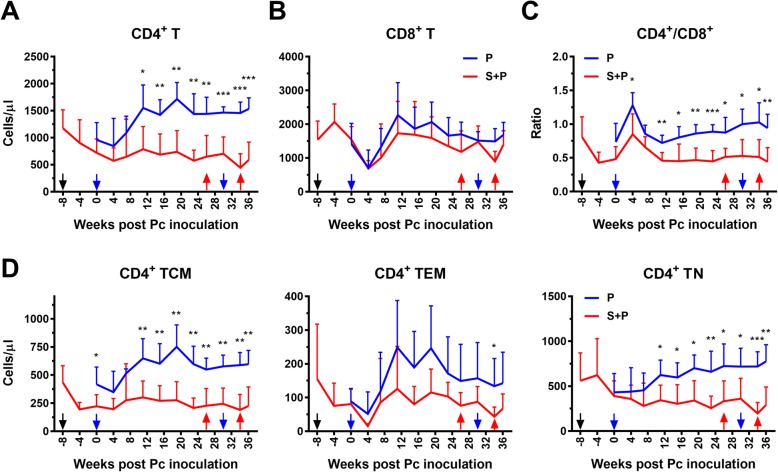
Responses of T cells to SIV and Pc infection. **a** Number of CD4^+^ (left) and CD8^+^ (right) T cells in the peripheral blood. **b** Ratio of CD4^+^/CD8^+^ T cells and (**c**) numbers of CD4^+^ TCMs (left), CD4^+^ TEMs (middle), and CD4^+^ TNs (right). Pc inoculation (blue arrow) and chloroquine treatments (red arrow) are indicated. Data are presented as the mean ± SD. An unpaired t test was performed to analyze the difference between the means of the two experimental groups, and statistical significance is indicated with *(*P* < 0.05), **(*P* < 0.01), or ***(*P* < 0.001)

We then analyzed the responses of CD4^+^ and CD8^+^ T cells during the course of SIV and Pc infection. The total CD4^+^ T cell count was decreased in the P group during the first 4 weeks of Pc infection and was then increased to high levels at week 11 and maintained during the remaining course of infection. In contrast, the total CD4^+^ T cell count of S + P animals decreased continuously after SIV infection. Following Pc infection, the CD4^+^ T cell count of S + P animals did not increase, but the CD4^+^ T cell count of animals in the P group did increase during the chronic phase of Pc malaria (Fig. 4a). The CD8^+^ T cell responses of the two groups of animals showed similar patterns, and no significant difference was observed between the two groups during the course of the experiment (Fig. 4b). As a result, the changes in the CD4^+^/CD8^+^ ratio in the two groups of animals during the course of SIV and Pc infection were comparable to the changes in the CD4^+^ T cell response (Fig. 4a, c).

Further analysis of the response of CD4^+^ subpopulations (Additional file 4: Figure S4) showed that the changes in the number of central memory T cells (TCMs, defined as CD4^+^CD28^high^CD95^high^) showed a pattern similar to those of the total CD4^+^ T cells. The animals in the S + P group had significantly weaker CD4^+^ TCM responses than the animals in the P group over most of the duration of the coinfection (Fig. 4d). CD4^+^ effector memory T cells (TEMs, defined as CD4^+^CD28-CD95^+^) responded to Pc infection in the two groups of animals in a similar manner, and no significant difference was observed between the two groups (Fig. 4d). CD4^+^ naïve T cells (TNs, defined as CD4^+^CD28^low^CD95^−^) increased following Pc infection in the P group animals, but they decreased after SIV infection in S + P animals and did not increase after Pc infection. The TN cell count in the P group animals was significantly higher than that in the S + P group animals from weeks 11 to 36 of Pc infection (Fig. 4d).

### Immune activation and CD4^+^ T cell dynamics during the acute phase of pc infection

**Fig. 5:**
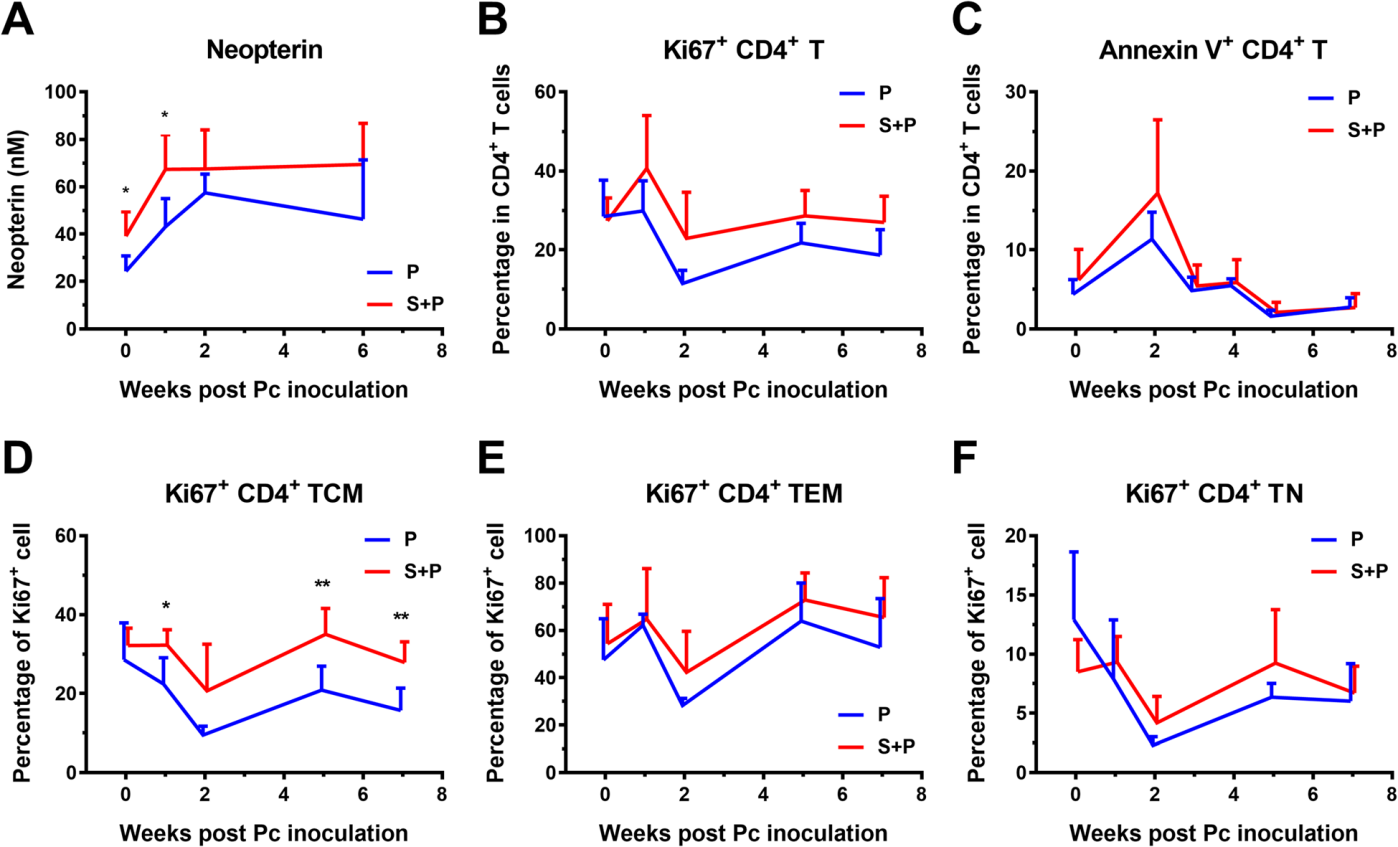
Activation and proliferation of CD4^+^ T cells during the acute stage of Pc malaria. **a** Neopterin concentration in the plasma. **b** The percentage of Ki-67^+^ cells among total CD4^+^ T cells. **c** The percentage of annexin V^+^ cells among total CD4^+^ T cells. The percentage of Ki-67^+^ cells among CD4^+^ (**d**) TCMs, (**e**) TEMs and (**f**) TNs. The data presented are the mean ± SD. Unpaired t tests were used, and statistically significant differences are indicated with *(*P* < 0.05) or **(*P* < 0.01)

**Fig. 6:**
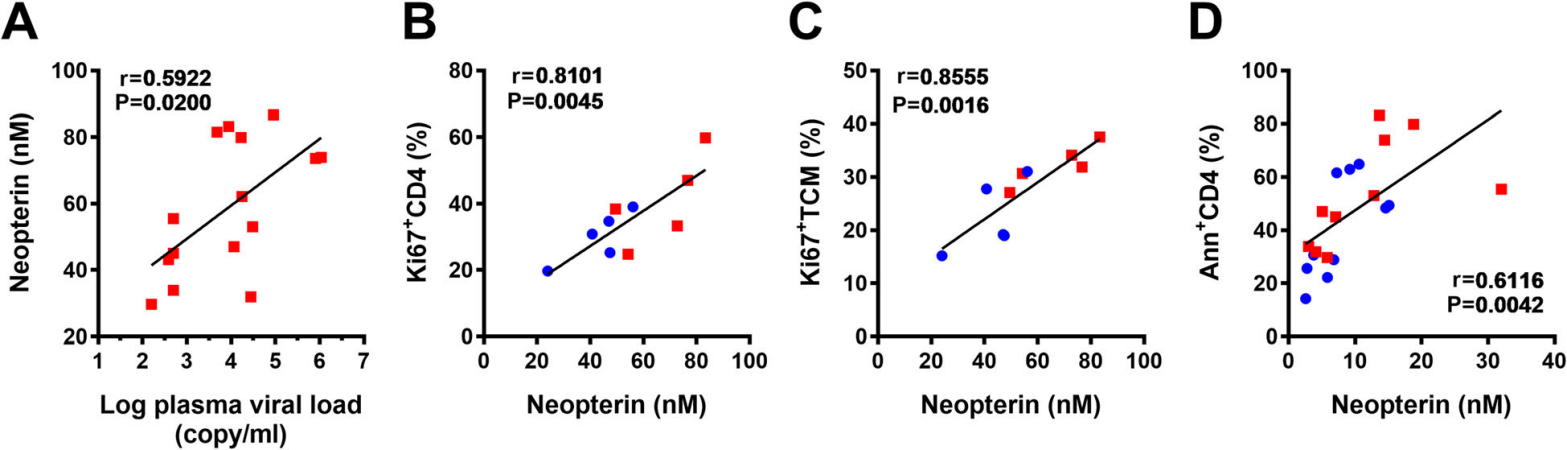
Analysis of correlations between neopterin and viral load or T cell activation. **a** Correlation between the logarithm of viral load and the neopterin concentration at weeks 0, 2 and 6. Correlation between the neopterin concentration and the percentage of CD4^+^Ki-67^+^ T cells (**b**) and the percentage of CD4^+^Ki-67^+^ TCMs (**c**) at week 1 of Pc infection. **d** Correlation between the neopterin concentration and the percentage of annexin V^+^ CD4^+^ T cells at weeks 0 and 2 of Pc infection. Linear regression analysis was performed, and the Pearson r values and P values are shown

Neopterin is recognized as a surrogate marker of Th1 immune activation. The animals in the S + P coinfection group produced higher levels of neopterin in the plasma than the normal animals before Pc infection (week 0, *P* = 0.0263) and 1 week after Pc infection (Fig. 5a, Additional file 5: Figure S5A). We then examined CD4^+^ T cell dynamics, including activation, proliferation and apoptosis. Ki-67 is recognized as a marker for T cell activation and proliferation, and annexin V is recognized as a marker for T cell apoptosis. We found that the frequencies of Ki-67^+^ cells among CD4^+^ T cells, TCMs, TEMs and TNs and the frequency of annexin V^+^ CD4^+^ T cells were higher in the S + P group animals than in the P group animals (Fig. 5b-f, Additional file 5: Figure S5B-F); however, only the frequency of Ki-67^+^ T cells among CD4^+^ TCMs was significantly different between the two groups (*P* < 0.05, *P* < 0.01, and *P* < 0.05 at weeks 2, 5 and 7, respectively, after Pc inoculation) (Fig. 5c, Additional file 5: Figure S5C). Further analysis revealed that the neopterin levels in the plasma were positively correlated with the plasma viral load from weeks 0 to 6 post-Pc inoculation (Fig. 6a). The frequency of Ki-67^+^ cells among total CD4^+^ T cells and among the CD4^+^ TCM subset was positively correlated with the neopterin concentration (Fig. 6b, c). The frequency of CD4^+^ T cell apoptosis was positively correlated with the neopterin concentration (Fig. 6d).

## Discussion

We established a nonhuman primate SIV and Pc malaria coinfection model to investigate the influence of concurrent SIV infection on the pathogenesis of malaria and the associated immunological responses under defined experimental conditions. We observed that compared with the animals subjected to Pc infection alone, Chinese rhesus monkeys with pre-existing SIV infection exhibited aggravated malaria symptoms following infection with the Pc malaria parasite. The S + P coinfected animals experienced longer episodes of malaria with higher peak levels of parasitemia, developed more severe anemia, and experienced greater weight loss and a slower recovery. In addition, the coinfected animals developed impaired CD4^+^ T cell responses and suppressed B cell proliferation and antibody production in response to primary Pc infection. Furthermore, the activation and apoptosis of CD4^+^ T cells were higher in S + P coinfected animals than in animals subjected to Pc infection alone. These impaired humoral and cellular immune responses resulting from the imbalanced immune activation in coinfected animals may contribute to the reduction of protective immunity to a level that is unable to efficiently control primary or secondary Pc malaria infection.

Animals infected with SIV or Pc in our model developed symptoms and disease progression that were similar to those of HIV infection or malaria in humans. The animals coinfected with Pc during chronic SIV infection experienced rapid parasite growth, which we had to control with several low doses of artesunate treatment to prevent animal from death. This low dose of artesunate cannot eliminate the parasites. After the treatment(s), parasites continued to replicate for several weeks in both groups and finally suppressed by host immunity. These observations indicated that the low doses of artesunate treatment in this study did not affect the results fundamentally. Furthermore, despite the drug treatment, the coinfected animals experienced more severe anemia and greater weight loss and took a longer time to recover from anemia and weight loss than the animals subjected to infection with malaria alone. These characteristics were very close to those of *P. fragile*-SHIV coinfected animals [24] or patients with HIV and malaria coinfection [5, 25, 26]. Our results were consistent with observations in humans that HIV-positive patients lacking immunity to malaria were more likely to suffer from severe malaria than HIV-negative individuals.

In addition to investigating the impact of SIV infection on primary Pc malaria, we further analyzed the effect of concurrent SIV infection on secondary Pc malaria. After clearing the primary malaria with chloroquine, the animals were reinfected with Pc parasites to initiate secondary malaria. We found that the animals in the P group exhibited immune protection and control of secondary Pc malaria, which was similar to the naturally acquired partial immunity reported in human adults in malaria endemic areas [27]. However, the animals in the S + P group developed mild clinical episodes with high levels of parasitemia. These results demonstrate that SIV infection acts as a risk factor for severe malaria. It has been reported that human patients who are naïve to malaria and infected with HIV are more likely to display severe malaria than HIV-negative persons. However, in individuals who are semi-immune to malaria, no significant difference in the risk of severe malaria was found between the HIV-infected and HIV-negative individuals [26]. Our observations show that SIV-positive animals developed more severe malaria symptoms during both primary and secondary Pc infection. This inconsistency is likely due to variations in the interval between the two infections and differences in the immune status of clinical patients.

The humoral antibody response is an important part of the immune effector mechanism against malaria. Studies in humans and monkey models demonstrated that concomitant HIV/SIV infection impairs anti-malarial antibody production [10, 11, 14]. Our results showed that Pc malaria infection induced robust proliferation of B cells and a strong antibody response in animals in the P group. These humoral immune responses were suppressed in S + P coinfected animals. HIV infection can directly impair B cell responses by exhausting memory B cells [28]. Furthermore, parasite infection can exacerbate this expansion of atypical memory B cells in patients coinfected with HIV and malaria [14]. Our results demonstrated that the S + P animals with a higher SIV viral load on the day of Pc inoculation produced lower levels of anti-Pc antibodies. Pre-existing SIV infection during primary Pc infection may impair antibody production, resulting in less effective immune protection for the control of both primary and secondary Pc malaria.

CD4^+^ T cells are essential for cytokine production, B cell activation and antibody production and phagocyte activation. Our results showed that the CD4^+^ T cell response to Pc malaria was significantly impaired in the S + P group animals, but the CD8^+^ T cell response was not affected. As observed by others [11], in our study, the numbers of TCMs and TNs decreased after SIV infection and showed a weaker response to Pc infection in the S + P group animals. A continuous decline in TCMs and TNs may result in delayed replenishment of exhausted TCMs or TEMs [29, 30]. Short-lived CD4^+^ TEM cells are believed to play a key protective role in controlling parasitemia [30–32]. We noticed in our study that the change in CD4^+^ TEM numbers was inversely associated with parasite density in both groups. Although this difference was not statistically significant, a higher number of CD4^+^ TEMs was detected in the P group animals than in the S + P animals. These TEMs may contribute to immune protection against Pc parasites during primary and secondary infection. We propose that the rapid exhaustion of CD4^+^ TEMs and the lack of replenishment of TEMs from the TN or TCM pool may lead to compromised immune protection against Pc malaria in S + P coinfected animals.

In this study, we evaluated Th1 immune activation by measuring the level of neopterin in the plasma during the acute stage of Pc malaria infection. We found that the levels of neopterin in both groups of animals increased following Pc inoculation. The higher level of neopterin in S + P animals on the day of Pc inoculation indicated that the SIV-infected animals were in a state of immune activation. The S + P animals maintained higher levels of neopterin during the acute phase of Pc infection. Chronic immune activation in HIV infection is one reason for the persistent decline of CD4^+^ T cells due to senescence and apoptosis, and eventually death, of these cells. We further analyzed the data on CD4^+^ T cell dynamics, including activation, proliferation and apoptosis. The frequencies of Ki-67^+^ among CD4^+^ T cells, TCMs, TEMs and TNs and the frequency of annexin V^+^ CD4^+^ T cells were higher in the S + P group than in the P group, even though only the difference in the frequency of Ki-67^+^CD4^+^ TCMs was statistically significant (Fig. 5). Unlike mouse studies, which can be easily repeated or which can increase the number of animals to examine whether the absence of statistical significance is due to an insufficient number of animals, we could not easily repeat our monkey model study or increase the number of animals. Therefore, we further analyzed the correlations among the above parameters related to CD4^+^ T cell dynamics based on significant differences in neopterin levels and Ki-67^+^CD4^+^ TCM frequency between the two groups. The analysis indicated that these parameters were correlated with each other (Fig. 6), suggesting that a rapid turnover of CD4^+^ T cells also existed in our S + P group animals. Therefore, SIV infection in S + P animals may result in aberrant immune activation and disease progression, as reported in HIV^+^ humans and SIV-infected macaques or sooty mangabeys [33–37], and eventually lead to an impaired immune response to Pc malaria.

## Conclusion

Overall, the present study demonstrated that SIV infection acted as a risk factor for primary and secondary *Plasmodium* infection. Severe malaria in coinfected animals might result from impaired humoral immunity and CD4^+^ T cell responses against parasites. These observations suggest that appropriate HIV therapy may be needed not only to alleviate clinical malaria in HIV-positive patients but also to reduce the risk of plasmodium parasite spreading in areas with a moderate or high prevalence of HIV and malaria.

## Supplementary information

**Additional file 1: Figure S1.** Parasitemia levels of individual monkeys in the P and S + P groups. Parasitemia was measured daily by blood smears. Pc inoculation, chloroquine and artesunate treatment are indicated by blue, red and green arrows, respectively.

**Additional file 2: Figure S2.** Body temperature changes in the individual animals in the P and S + P groups. Body temperature was measured daily. The mean body temperature of each animal measured from day − 7 to 0 of Pc infection is shown by a horizontal dotted line. One SD and 2 SDs of the mean body temperature are indicated by dark gray and light gray zones, respectively. A body temperature higher than 2 SDs of the mean was considered a fever.

**Additional file 3: Figure S3.** (A) The area under the curve (AUC) of temperature during fever. (B) The duration of fever. (C) The difference in the greatest body weight change between the two groups.

**Additional file 4: Figure S4.** Gating strategy used in flow cytometric analysis to detect different memory subsets of circulating CD4^+^ T cells. Sample data are shown. (A) Lymphocytes were gated on a forward scatter (FSC)/side scatter (SSC) plot. (B) Lymphocytes were then further gated to determine CD4^+^ and CD8^+^ cells. (C) CD4^+^ cells were further gated to determine TNs, TCMs and TEMs.

**Additional file 5: Figure S5.** Activation and proliferation of CD4^+^ T cells during the acute stage of Pc malaria and SIV infection. (A) Neopterin concentration in the plasma. (B) The percentage of Ki-67^+^ cells among total CD4^+^ T cells. (C) The percentage of annexin V^+^ cells among total CD4^+^ T cells. The percentage of Ki-67^+^ cells among CD4^+^ (D) TCMs, (E) TEMs and (F) TNs. The data presented are the mean ± SD. Unpaired t tests were used, and statistically significant differences are indicated with *(*P* < 0.05) or **(*P* < 0.01).

## Data Availability

The datasets used in the current study are available from the corresponding author on reasonable request.

## Abbreviations

AIDS: acquired immunodeficiency syndrome
HIV: human immunodeficiency virus
i.v.: intravenously
iRBCs: infected red blood cells
Pc: *Plasmodium cynomolgi*
SIV: simian immunodeficiency virus
TCID_50_: median tissue culture infectious dose
TCMs: central memory T cells
TEMs: effector memory T cells
TNs: naïve T cells
WHO: World Health Organization
