# Supplementary figures and images for: SIV infection aggravates malaria in a Chinese rhesus monkey coinfection model
Source: BMC Infect Dis. 2019 Nov 12;19:965. doi: 10.1186/s12879-019-4465-6 (PMC6852750; doi:10.1186/s12879-019-4465-6)

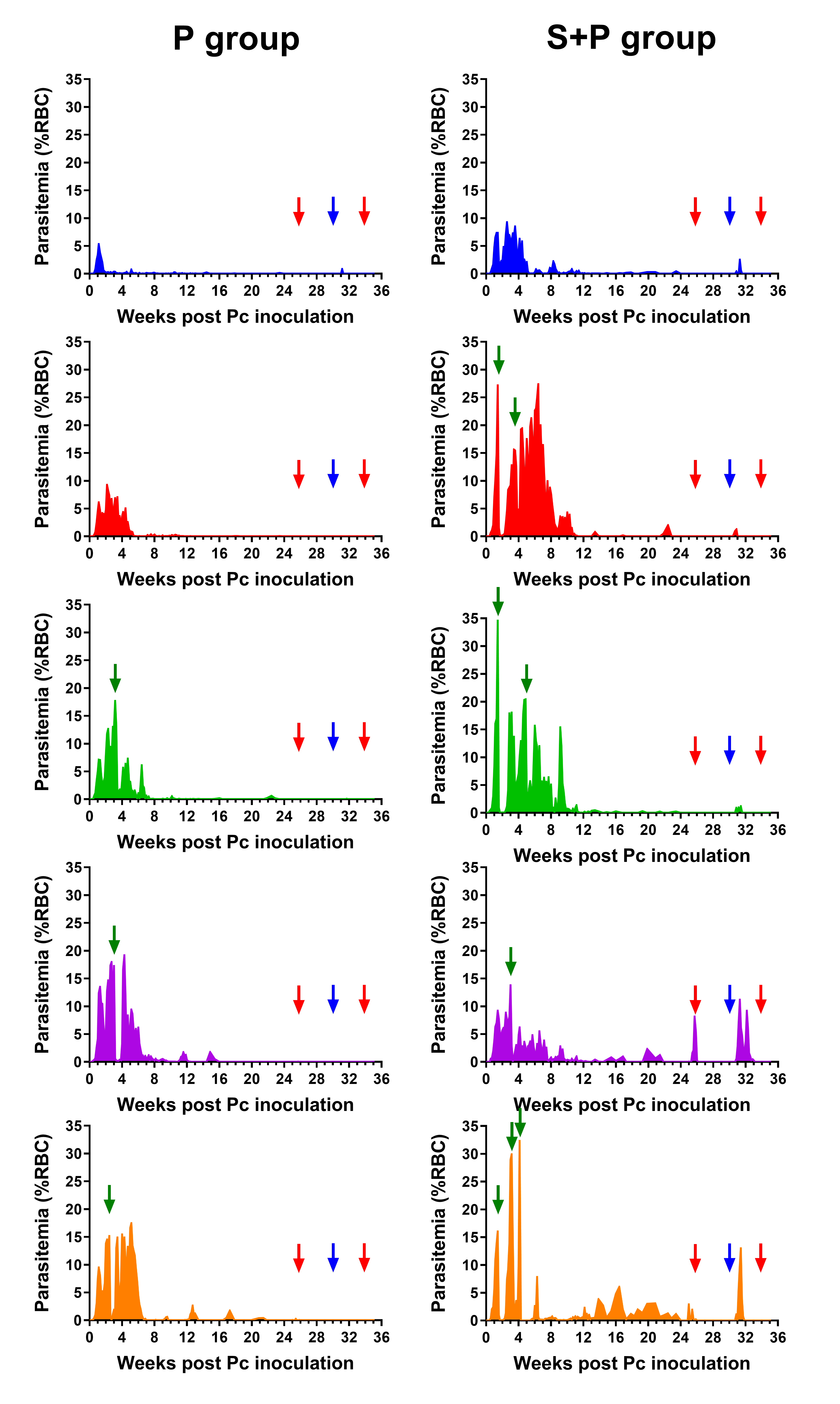

Supplement: Supplementary file 1 — Additional file 1: Figure S1. Parasitemia levels of individual monkeys in the P and S + P groups. Parasitemia was measured daily by blood smears. Pc inoculation, chloroquine and artesunate treatment are indicated by blue, red and green arrows, respectively. [file 12879_2019_4465_MOESM1_ESM.tif]

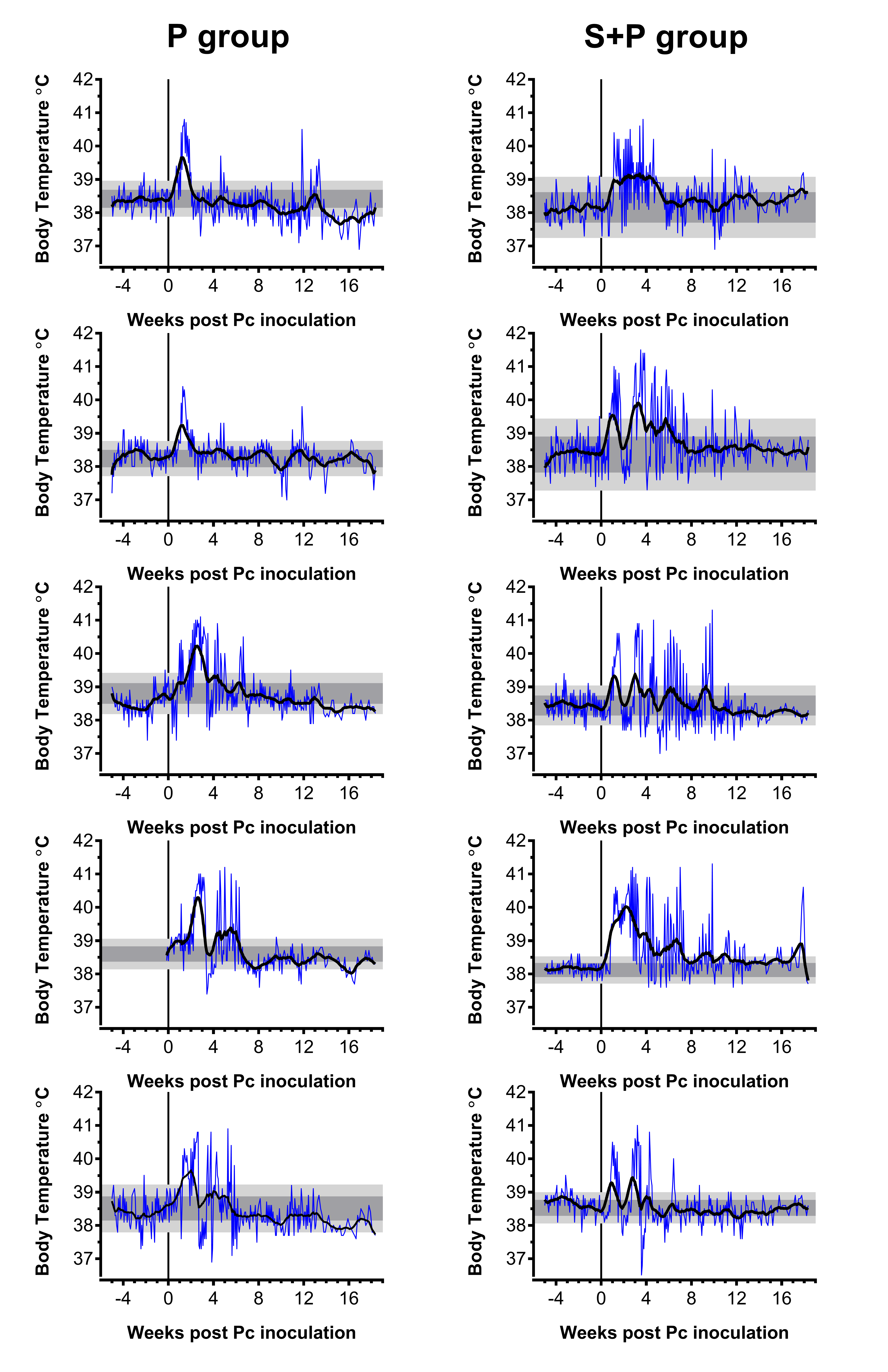

Supplement: Supplementary file 2 — Additional file 2: Figure S2. Body temperature changes in the individual animals in the P and S + P groups. Body temperature was measured daily. The mean body temperature of each animal measured from day − 7 to 0 of Pc infection is shown by a horizontal dotted line. One SD and 2 SDs of the mean body temperature are indicated by dark gray and light gray zones, respectively. A body temperature higher than 2 SDs of the mean was considered a fever. [file 12879_2019_4465_MOESM2_ESM.tif]

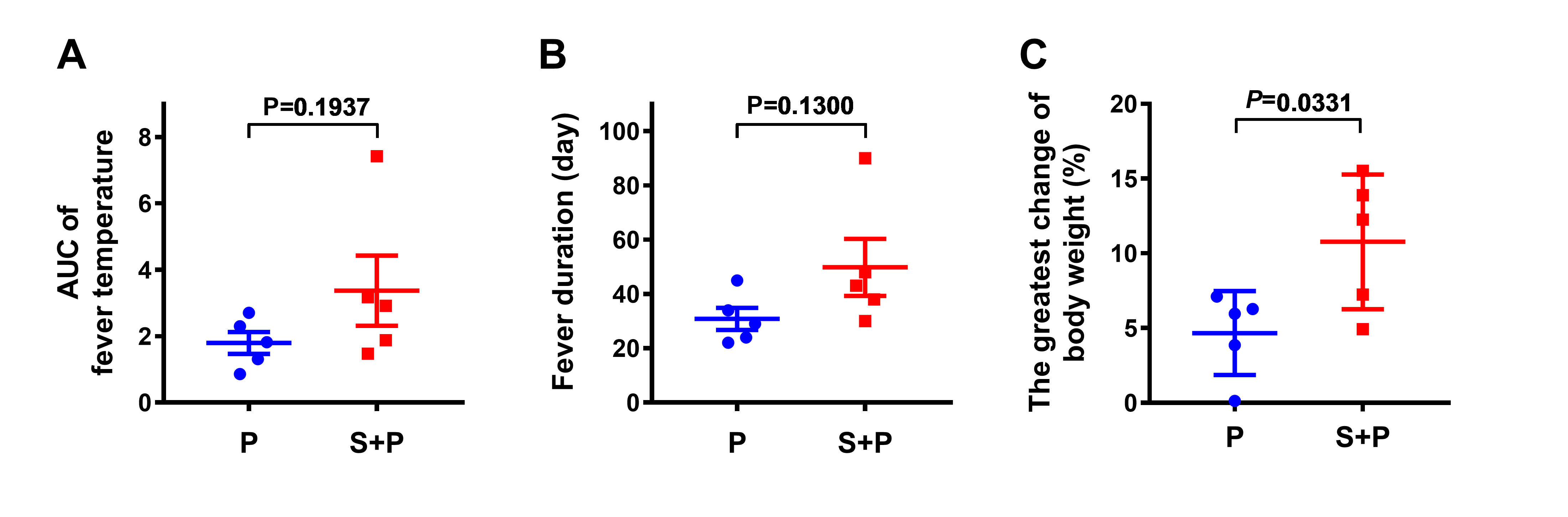

Supplement: Supplementary file 3 — Additional file 3: Figure S3. (A) The area under the curve (AUC) of temperature during fever. (B) The duration of fever. (C) The difference in the greatest body weight change between the two groups. [file 12879_2019_4465_MOESM3_ESM.tif]

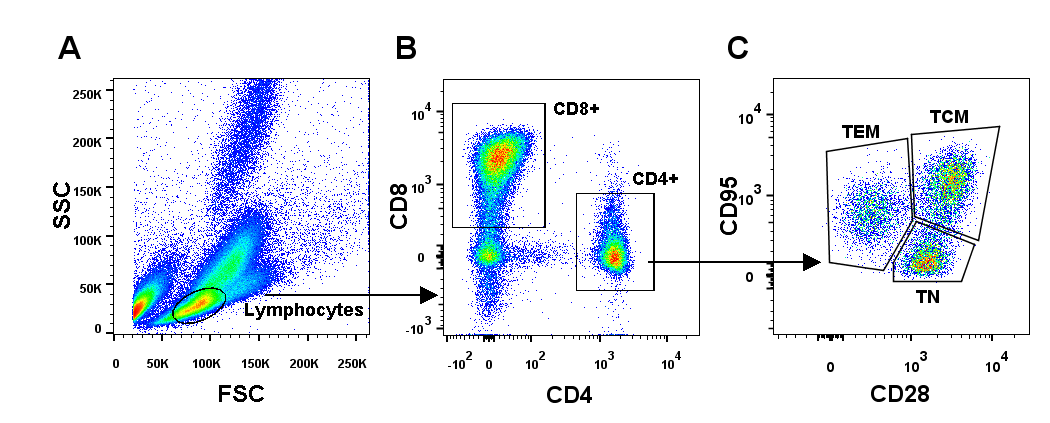

Supplement: Supplementary file 4 — Additional file 4: Figure S4. Gating strategy used in flow cytometric analysis to detect different memory subsets of circulating CD4+ T cells. Sample data are shown. (A) Lymphocytes were gated on a forward scatter (FSC)/side scatter (SSC) plot. (B) Lymphocytes were then further gated to determine CD4+ and CD8+ cells. (C) CD4+ cells were further gated to determine TNs, TCMs and TEMs. [file 12879_2019_4465_MOESM4_ESM.png]

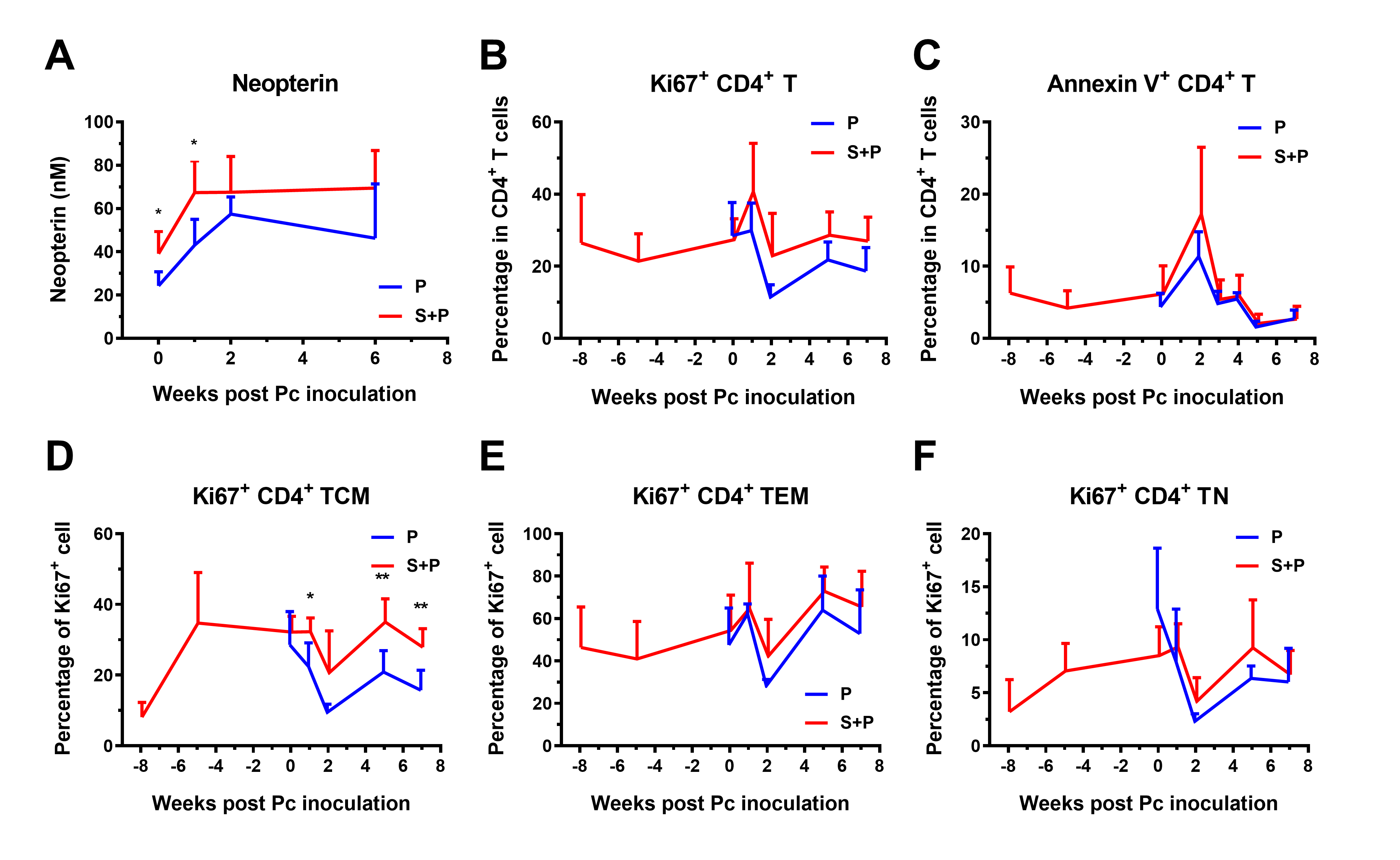

Supplement: Supplementary file 5 — Additional file 5: Figure S5. Activation and proliferation of CD4+ T cells during the acute stage of Pc malaria and SIV infection. (A) Neopterin concentration in the plasma. (B) The percentage of Ki-67+ cells among total CD4+ T cells. (C) The percentage of annexin V+ cells among total CD4+ T cells. The percentage of Ki-67+ cells among CD4+ (D) TCMs, (E) TEMs and (F) TNs. The data presented are the mean ± SD. Unpaired t tests were used, and statistically significant differences are indicated with *(P < 0.05) or **(P < 0.01). [file 12879_2019_4465_MOESM5_ESM.tif]
